# Attitudes and Mental Files in Discourse Representation Theory

**DOI:** 10.1007/s13164-015-0296-6

**Published:** 2016-01-12

**Authors:** Emar Maier

**Affiliations:** Department of Philosophy, University of Groningen, Oude Boteringestraat 52, 9712GL Groningen, The Netherlands

## Abstract

I present a concrete DRT-based syntax and semantics for the representation of mental states in the style of Kamp (1990). This system is closely related to Recanati’s (2012) Mental Files framework, but adds a crucial distinction between anchors, the analogues of mental files, and attitudes like belief, desire and imagination. Attitudes are represented as separate compartments that can be referentially dependent on anchors. I show how the added distinctions help defend the useful notion of an acquaintance-based mental file against Ninan’s (*Inquiry* 58(4):368–377 2015) recent challenge involving counterfactual *de re* attitudes.

## Introduction

In a previous special issue on Mental Files, Ninan (2015) argues that non-doxastic attitudes challenge Recanati’s (2012) account. His counterexample runs as follows. Lucy does not realize that Samuel Clemens is Mark Twain and believes that Twain is famous but Clemens is not. This is just a basic Frege puzzle: Lucy’s beliefs are *de re* and involve distinct modes of presentation of a single *res*. On Recanati’s view this means Lucy has two distinct mental files that both refer to the same individual. Lucy’s Clemens-file will have the predicate IS NOT FAMOUS inscribed in it, while her Twain-file contains IS FAMOUS. Now, Ninan observes, this mental files analysis of Frege cases does not extend to counterfactual attitudes like imagination. Say, Lucy imagines that things were the other way around, i.e. Samuel Clemens is famous but Mark Twain is not. Again, we’re dealing with two incompatible *de re* attitudes about the same individual, so these attitudes must again involve distinct Clemens- and Twain-files. But how can Lucy’s Clemens-file, which contains IS NOT FAMOUS, capture her mental state of imagining that he is famous?

Apparently there is an asymmetry between the attitudes of belief and imagination in the way the attitudinal contents relate to what’s in the files. Ninan then, helpfully, suggests a possible way for Recanati to deal with this:

Perhaps Recanati has a different picture in mind. Maybe Lucy’s mind can be thought of as divided up into boxes corresponding to the different types of attitudes that she has: there is a belief box, a desire box, an imagination box, etc. Lucy’s belief box contains a Clemens-file, as does her imagination box (perhaps these two files are in some way linked). If Lucy imagines that Clemens is famous and Twain is not, then the Clemens-file in her imagination box has the predicate IS FAMOUS inscribed on it, even though the Clemens-file in her belief box has the predicate IS NOT FAMOUS inscribed on it. The predicates inscribed on the files in her belief box are irrelevant to the content of her imagining. (Ninan 2015:376)

Recanati’s (2015) reply shows that he does indeed think an analysis along these lines–supplemented with an account of file-linking in terms of generalized indexing–may be among the viable solutions. But Ninan ends on a pessimistic note:

Perhaps some such story could be told. But note one upshot of extending the account in this way: one can no longer characterize mental files as mental representations whose primary function is to carry information about objects. (Ninan 2015:376)

In this paper I show that “some such story” can and has been told, viz. by Kamp (1990, …,^2015^), Asher (1986, 1987), and myself (2015b, 2015c, 2016) working in the framework of Discourse Representation Theory (DRT). Moreover, I will show how the DRT analysis of compartmentalized mental states allows us to “characterize mental files as mental representations whose primary function is to carry information about objects.”

The structure of the paper is as follows. I first introduce basic DRT (Section 2) and then reconstruct a version of the DRT-based representational theory of mental states that I will refer to as Attitude Description Theory (ADT) (Section 3). I demonstrate the ADT framework by applying it to some philosophically interesting phenomena, paying special attention to any differences between Kamp’s ADT and Recanati’s Mental Files (Section 4). I then present a more precise formalization of the syntax and semantics of ADT (Section 5). I end by showing how ADT deals with Ninan’s challenge (Section 6).

## Discourse Representation Theory

Discourse Representation Theory (DRT, Kamp & Reyle 1993) is typically presented as a specific type of formal semantics, well suited for dealing with semantics/pragmatics interface phenomena like presupposition and anaphora resolution. DRT’s formal language of Discourse Representation Structures (DRSs) is then used to represent the Stalnakerian common ground between speaker and hearer. The explanatory power of the framework lies in the algorithms for updating these common ground representations in response to linguistic utterances.

To illustrate the basic DRT framework, consider (1).

1. A woman is standing outside.

In DRT, utterances are interpreted as updates on a context, represented as a DRS. Let’s assume we’re starting with an empty context DRS when we hear (1). The indefinite *a woman* introduces a new discourse referent into the common ground. Concretely, interpreting the sentence transforms the empty input DRS into the following output DRS:

(2)

This boxy formula is read as ‘there exists an x that has the properties of being a woman and of standing outside’. In other words, with the standard syntax and semantics of the DRS language (cf. Kamp & Reyle, 1993), (2) is truth-conditionally equivalent to (and recursively translatable into) the first-order formula ∃x[woman(x) ∧ stand.outside(x)].^1^ To illustrate the dynamic character of the theory, let’s say that the speaker of (1) continues with (3).

(3) She’s smoking.

The subject of this sentence is a pronoun, *she*, which is treated not as introducing new material but as looking for a previously established (“familiar”) discourse referent to bind to. In this case, the pronoun, initially represented as ‘?’, can be bound to the discourse referent x that was introduced into the common ground by the previous sentence:

(4)

The final output represents the truth-conditions of the whole two-sentence discourse: there’s a woman standing outside smoking.

The analysis of anaphora has been extended significantly to provide, for instance, one of the leading theories of presupposition van der Sandt (1992) and Geurts (1999). Other areas where DRT shines include quantification, tense and aspect, and propositional attitude ascriptions.

What is often glossed over in such linguistic applications – even in many analyses of attitude ascriptions, including my own 2010 – is Kamp’s (1981) original motivation of reconciling Fregean formal semantics (as championed at the time by Montague (1973)) with a traditional, Lockean cognitive theory of communication in terms of speakers’ and hearers’ mental states. To this end, Kamp in his original presentations actually describes DRSs as representations of the mental state of the hearer, rather than of the more abstract notion of a Stalnakerian common ground. What sets this cognitive conception of DRT apart from purely cognitive theories like Fauconnier’s (1994) Mental Spaces, is that the DRS language has a precise syntax and model-theoretic interpretation. Hence, in addition to its cognitive interpretation, a DRS also represents the actual truth conditions of a sentence or discourse.

Linguists have since stripped DRT of its cognitive interpretation. But Kamp and a few others have kept it alive, even extending DRT to a full-blown representational theory of attitudes (Kamp 1990; Asher 1986). In the following sections I present a version of such a DRT-based theory of mental states known as Attitude Description Theory (ADT).

## Anchors and Attitudes

The starting point of ADT is that mental states are (i) compartmentalized into beliefs, desires, fears, intentions, etc., and (ii) these compartments are highly interconnected. For instance, my mental state could contain the belief that there’s a monster under my bed and, dependent on that belief, the hope that *it* won’t wake up. This dependence is cashed out in the same way as anaphoric dependencies in discourse are modeled in standard DRT, viz. by sharing accessible discourse referents.

To model singular attitudes, Kamp further introduces the notion of “entity representations” or “internal anchors”. These internal anchors contain descriptive information about the way we are acquainted with particular objects in the world around us. An anchor thus serves as a cognitive mode of presentation of the object that is the causal source of a certain *de re* attitude. Kamp’s internal anchors are reminiscent of what philosophers have called dossiers (Grice 1969), or mental files (e.g. Perry 1980; Recanati 2012). In the following I will stick with the term *anchor*, and note where Kamp’s and Recanati’s accounts diverge.

By way of illustration of the theory thus far, the so-called Attitude Description Set (ADS) below represents the mental state I’m in when I look in my pigeon hole at work and see an envelope, believe it’s publisher’s junk mail, hope it’s a letter of acceptance for a recent grant application, and intend to open it right away.^2^

(5)

This ADS contains at the global level two perceptual anchors, representing my perceptual acquaintance with the letter and the mailbox it’s in. Formally, an anchor consists of a label (anch) and a DRS that introduces a new anchored discourse referent along with the acquaintance-based descriptive mode of presentation that the agent associates with it.^3^

Below the anchors we find three attitude descriptions (a belief, a desire, and an intention), again consisting of a label (bel, des, int) and a DRS that gives the descriptive content of the attitude. A key feature of the framework is that discourse referents may be shared across anchors and attitudes. In our example the first anchor uses a discourse referent introduced in the second, and all three attitudes use the discourse referent x. We’ll say that these attitudes referentially depend on the anchor for the letter, which in turn referentially depends on the anchor for the mailbox. Finally, to model *de se* attitudes there is a special, essentially indexical discourse referent i, which we may think of as introduced by an implicit, non-descriptive anchor that represents the agent’s self (cf. Section Section 4.1).

The actual causal sources of internal anchors are represented outside of the ADS proper. Kamp formalizes the causal links between a mental state and its surroundings as a mapping from discourse referents to entities. In our example, this so-called “external anchor” maps x to the actual letter, y to the mailbox, and i to me:

(6) $\left [\begin {array}{ll} \text {x} & \mapsto \text {letter}\\ \text {y} & \mapsto \textnormal {mailbox}\\ \text {i} & \mapsto \text {Emar} \end {array}\right ]$

The external anchor allows us to capture singular attitudes: any attitude compartment that depends on (i.e., makes use of) a discourse referent introduced by an externally anchored internal anchor represents a genuinely singular attitude, about the causal source of that internal representation. In our example all three attitudes, the belief, the hope, and the intention depend on the anchor introducing x, representing the letter as something I am seeing in my mailbox. Consequently, they are *about* the causal source of that mental representation, the actual letter.

Before I go into the formal semantics in Section 5, let me further illustrate the power of the representational framework by applying it to some more interesting phenomena and scenarios from the philosophical literature.

## Case Studies

### Attitudes *de se*

We use the dedicated indexical discourse referent i to represent the subject’s first person *de se* center. Similarly, we could use n to model the subject’s subjective now, but for simplicity I’ll ignore the temporal domain throughout. In (7) we see a fragment of the mental state of Lingens who self-ascribes the property of being a lost amnesiac, while reading about Lingens in an encyclopedia and learning that this Lingens is working in Stanford.

(7)

The crucial realization that follows this state of confusion in Perry’s story, that he himself is Lingens, can then be represented by adding the condition ‘x =i’ to the belief box in (7).

Note that there is no explicit anchor introducing the discourse referent i in (7). The idea is that, since we’re assuming that every mental state contains a unique, non-descriptive, first person representation of the self we can suppress such an anchor in our ADS notations.

The implicit self-anchor is like other anchors in that it introduces a discourse referent that is thereby accessible to all attitudes and other anchors. However, Kamp (2011) assumes a fundamental distinction between *de re* and *de se*, based on the idea that subjects have a privileged, direct access to themselves, ‘unencumbered by any form of descriptive content’ (p.8) and ‘immune to error through misidentification’ (p.51) (i.e., when I self-ascribe being a linguist there can be no mistake that the person I’m ascribing this property to is me, but when I ascribe being a linguist *de re* to the person I see in the mirror I might be confused about who it is, or even whether there is anyone there at all). More on hallucination and faulty perception in Section 4.3, but I should note here already that on this point Kamp’s view diverges from that of Recanati, who pleads for of a more uniform approach to *de re* and *de se* attitudes based on the idea that the actual reference of a mental file is *always* fully independent of its descriptive content, and hence that *de re* reference is always ‘direct’ and ‘unencumbered’ by any (accurate or inaccurate) descriptive information. Pending a more thorough exploration of this disagreement over the *de re*/*de se* distinction, I will follow Kamp in this paper. In Section 5.3 I will cash out the special status of the self semantically by treating i as an indexical that picks out the center of any doxastic alternative of the agent.^4^

### Double Vision

The philosophical literature on attitudes and ascriptions is replete with puzzles where an agent forms two distinct representations of something, failing to realize that it is actually the same thing: Frege’s Babylonians see a distinct morning and evening star, Quine’s Ralph sees mayor Ortcutt as distinct from the suspicious figure in the alley, Kripke’s Pierre believes London is terrible, but Londres is pretty, Perry’s shopper believes himself to be distinct from the shopper with the torn sack, etc. In the current framework, each of these cases involves a mental state description with two distinct files, each associated with different descriptive contents based on the different ways of being acquainted with an entity, but the external anchor maps both files to the same real-world entity, the actual causal source.

Pierre’s predicament, for instance, can be represented as follows (Kripke 1979):

(8)

In Pierre’s mental representation of the world there are two distinct cities: one is the city he read about in his childhood in France, called *Londres*; the other is the city he lives in, called *London*. Based on these two epistemic links, he has formed two mental files, and through these he can have singular beliefs, hopes, etc.. We, as outside observers, know that his beliefs are in fact inconsistent. As represented in (8) by the external anchor, both mental files derive from a single source, so both beliefs are in fact *de re* about the same city. However, intuitively, the fact that Pierre believes the one city to be pretty and the other not does not entail that Pierre himself is irrational in the sense that his internal mental state is logically inconsistent.

To reconcile this apparent contradiction I will provide in Section 5 a model-theoretic semantics for mental state descriptions that defines both a narrow and a wide content of attitudes. The narrow content of Pierre’s beliefs as represented in (8) should be computed on the basis of the descriptive conditions in the belief box and the internal anchors on which those depend. More specifically, the narrow belief content expressed by (8) should be the (centered) proposition that the city the subject knows as ‘Londres’ is pretty and the city he knows as ‘London’ is not. This gives us the non-contradictory interpretation that captures what goes on in Pierre’s head, and that should explain his actions and practical reasoning. By contrast, the wide content of Pierre’s beliefs is computed by evaluating the same belief conditions, but relative to the external anchor, bypassing the descriptive content in the internal anchors. Computing the wide content of (8) will give us the singular proposition about London that it is both pretty and not pretty, a genuine contradiction.

### Faulty Perception

So far we’ve mostly discussed anchors based on actual acquaintance relations. Formally, the internal anchors we’ve encountered were all externally anchored. Given that anchors are supposed to represent the objects of our *de re* attitudes, based on our acquaintance with our surroundings, this is as it should be. Hence, Kamp’s (2011) slogan: “no internal anchor without an external anchor”. But what if I merely hallucinated seeing the letter in my mailbox (in the scenario from Section 3)? My narrow mental state in such a scenario will be the same as before, i.e., I cannot distinguish between the two situations. But now there is no causal source, i.e., no external anchor for my internal anchor representation of the letter. Consequently, the narrow contents of my belief, desire, and intention, remain the same while no wide content is expressed by my attitudes (that I take to be) about the letter.

In order to accommodate such cases, I follow a suggestion from Recanati (2012) to the effect that the external grounding of all mental files constitutes a normative requirement: internal anchors should be, and hence can be expected to be, externally anchored. The agent, in any case, assumes all her internal anchors to be externally anchored. Thus, the internal anchor for the letter in my mailbox plays the same role inside my mental life regardless of whether it’s properly anchored or hallucinated.

Kamp goes one step further: if a mental file “has no external anchor corresponding to the representation’s internal anchor (i.e. there is no entity to which agent and representation are causally related *in the way the internal anchor describes*), then the internal anchor is ‘ungrounded”’ (Kamp 2011:5, emphasis added). In other words, not only does an internal anchor presuppose an external anchor, the descriptive content of the anchor needs to mirror precisely the actual causal relation between agent and *res*. As before, this extra matching requirement is best thought of as a normative ideal that, in reality, is not always achieved. In other words, the agent herself will always assume all her internal anchors to be externally anchored to individuals that actually exemplify the properties associated with them in the internal anchor DRSs.

Nonetheless, Kamp’s strong matching requirement imposes an important restriction on the kind of information that belongs to the internal anchor. Only information about the way I’m acquainted with the object, e.g. as “the rectangular object I see now right in front of me in my hands”, should go into the internal anchor. By contrast, information that I then gather or infer about or otherwise associate with that object should go into a belief box referentially dependent on such an anchor. Where to draw the exact line between these two types of content however is an open question – in fact it’s just the old question of what counts as a relation of acquaintance, or what are the conditions under which we form *de re* attitudes. Concretely, going back to my representation of the letter example (5) from Section 3, conditions like ‘see(i,x)’ and ‘in.front.of(i,x)’ indeed belong in the internal anchor, while ‘junk(x)’, representing my belief that the envelope contains junk mail, is correctly represented as a (*de re*) belief about the letter instead. But how about the information that what I see is an envelope, is made of paper, is white? Or the information that x bears a certain name, or that y uses a certain expression to refer to x (cf. Section 4.4)? Since a proper theory of acquaintance and names is beyond the scope of this paper I will leave this decision for a future occasion. For now I’ll continue to put information about both perceptual links and names in internal anchors.^5^

This brings us back to the divergence between Kamp and Recanati that we first came across in discussing the *de se* in Section 4.1. As I said there, Recanati sees the content of the file as being independent of the actual relation of acquaintance:

What [mental files] refer to is not determined by properties which the subject takes the referent to have (i.e. by information—or misinformation—in the file), but through the relations on which the files are based. The reference is the entity we are acquainted with (in the appropriate way), not the entity which best ‘fits’ information in the file. (Recanati 2012:35)

By contrast, we just saw that Kamp holds that the referent, i.e. the external anchor, should fit the descriptive information in the file, i.e. the internal anchor. Interestingly, this disagreement between Kamp and Recanati may well be related to Ninan’s argument, discussed in Section 1, and the way it is resolved in ADT, as we will see in Section 6. Since Recanati does not distinguish mental file content from beliefs (and other attitudes), his files must allow all the information that an agent associates with a *res*, not just the relational acquaintance information.

### Proper Names and Vicarious Anchors

Not all mental files derive from direct perception. For instance, I have a mental file on Aristotle that is based on book reading and hearing other people use the name. My Aristotle file is externally anchored to the actual philosopher Aristotle and this allows me to have *de re* beliefs and imaginations about him, despite the fact that I have no direct perceptual relation to this ancient philosopher. In this case, the “epistemically rewarding” acquaintance link between Aristotle and me is not direct, but involves a long causal–historical chain of communication (à la Kripke 1980).

Kamp (2015) introduces the notion of a *vicarious anchor* to describe such *de re* attitudes based on testimony with proper names.

A vicarious anchor is established by some agent H who is witness to an act of reference by another agent S, and who, on the basis of this, establishes an entity representation R for the referent of that act. The vicarious internal anchor of that representation is the mark of this referential intention on the part of H and it is what makes [the vicarious internal anchor] into a representation of that referent. (Kamp 2015, 283–284)

A vicarious anchor is like a regular perceptual anchor in that it signals a referential intention. It differs from a perceptual anchor in that it doesn’t directly refer to its source, but defers the interpretation to another agent. Vicarious anchoring thus allows the agent to have singular attitudes about some individual she has no direct perceptual acquaintance with, other than hearing someone use their name.

We capture vicarious anchoring – at least for names – with a condition ‘refer(x,y,z)’ (speaker x used expression y to refer to z). For instance, when my wife starts talking to me about how Susan was being obnoxious at work today and I cannot link this name to any of my existing internal anchors, I may introduce into my mental state a new, vicarious anchor to refer to Susan:

(9)

## A Model-Theoretic Interpretation

ADSs may give a pretty picture of, say, Pierre’s mental state, but what does it really mean to say that Pierre has a mental state as described in (8)? In what sense does Pierre’s mind contain a set of labeled DRSs?

### Possible Worlds and Attitudes

I take as my point of departure the familiar possible worlds conception of propositions and beliefs. A person’s beliefs are described by a set of possible worlds, her doxastic alternatives. We can explicate the notion of a doxastic alternative as follows: *w*^′^ is a doxastic alternative of *a* in *w* (notation: *w*^′^∈*Dox*(*a*, *w*)) means that if you take *a* from world *w*, freeze her mental state, and place her in world *w*^′^, she will not be able to tell the difference. We then say that this person believes the proposition that it is raining iff all her doxastic alternatives are worlds where it is raining: *a* believes proposition *p*(⊆*W*) in *w* iff *Dox*(*a*, *w*) ⊆ *p*.

In order to deal with the essentially indexical discourse referent i we switch from worlds and propositions to centered worlds (formalized as world–individual pairs) and centered propositions (i.e., sets of centered worlds), respectively. We take *Dox*(*a*, *w*) to denote a set of centered worlds, i.e. 〈*w*^′^, *a*^′^〉 ∈ *Dox*(*a*, *w*) means that if you place *a* in *w*^′^ and let her experience it from the perspective of *a*^′^, she will be unable to distinguish it from *w* as experienced from her own perspective (Lewis^1979^; Haas-Spohn 1994). We then formalize *de se* belief as follows: Lingens in *w* believes *de se* that he is lost (or: Lingens self-ascribes in *w* the property of being lost) iff *Dox*(*Lingens*, *w* )⊆ {〈*w*^′^, *a*^′^〉|*a*^′^ is lost in *w*^′^}.

As a first approximation, let’s assume the same story applies to other attitudes: to model desires we have *Bul*(*a*, *w*) denoting the set of *a*’s centered buletic alternatives in *w*, and for imagination we have a set of imagination alternatives. A person’s full mental state can thus be characterized as a sequence of subsets of *C*, the set of centered worlds. Formally, these attitude characterizations of people across possible worlds are part of the model, i.e., a model $\mathfrak {M}$ is a tuple 〈*D*, *W*, *I*,〈*Dox*, *Bul*, *Img*…〉〉 with *C* = *D*×*W*, and $Dox, Bul, Img, \ldots : C\rightarrow \mathcal {P}(C)$.

So how do we relate this Lewis/Hintikka-style set-theoretic conception of an agent’s various attitudes, to our syntactic, DRT-based mental state descriptions? The central definition of ADT semantics says that an ADS $\mathcal {K}$ is a correct (partial) description of *a*’s mental state in *w* iff the narrow contents of the belief, desire, imagination, etc. components within *K* are compatible with the corresponding sets of doxastic, buletic, imaginative etc. alternatives of *a* in *w*.

Making this precise, taking into account also the anchors in *K*, requires a model-theoretic interpretation of the various labeled parts of a mental state description in terms of centered worlds. This in turn requires an intensional semantics for standard DRT, which I sketch below.

### The Syntax and Semantics of Basic DRT

Let’s start with some syntactic terminology. A DRS consists of two compartments. The top compartment, *U*(*K*), the so-called universe, contains the discourse referents. The bottom part, *Con*(*K*) contains the conditions, which are either atomic formulas (e.g. see(y,x)), or complex ones containing subDRSs (e.g. ¬*K*^′^ or *K*^′^→*K*^″^).

The central notion of DRT semantics is that of a verifying embedding, which is a partial function from the set of discourse referents to the model’s domain *D*. For our current purposes we need an intensional parameter. Since we take doxastic and buletic alternatives to be centered worlds, we’re going to need those as our intensional parameter. We say that an embedding *g* verifies a DRS *K* in *c* iff it verifies all conditions of *K*:

(10) *g*⊧_*c*_*K* iff for all*ψ*∈*Con*(*K*):*g*⊧_*c*_*ψ*.

Condition verification is defined by cases. For example:

(11) $\begin {array}{ll} \text {a.}{\kern 4pt} g\models {c} P(x_{1},{\ldots } x_{n}) iff \langle g(x_{1}),\ldots ,g(x_{n})\rangle \in I_{c}(P)\\ \text {b.} {\kern 4pt} g\models {c} \neg K^{\prime } \text {iff there is no} h\supseteq \text {g}~\text {with}~ Dom(h) = Dom(g)\cup U(K^{\prime })~\text {and}\\ {\kern 11pt} h\models {c} K^{\prime }. \end {array}$

The essential indexical discourse referent i can be dealt with as a special case of “anchoring” as it was originally developed for standard DRT by Kamp and Reyle (1993). Like the later external anchors of ADT, an anchor here is just a partial embedding that fixes the reference of a number of discourse referents. For i we add a global stipulation that fixes its reference to *a*_*c*_, the agent parameter of the centered world of evaluation *c*. Our central definition of truth involves the truth of a DRS *K* in a centered world *c* relative to an anchor *f*:

(12) $[[ K]]^{f}_{c} = 1~ \text {iff there is an embedding}~ g\supseteq f \text {such that} ~Dom(g)=U(K)\cup \{\mathrm {i}\} \\ \text {and}g\models _{c} K, \text {and}, \text {moreover}, g(\mathrm {i})=a_{c}.$

From there we can define also the (centered) proposition expressed by *K* relative to *f* as the set of centered worlds in which *K* is true relative to *f*.

(13) $[[ K]]^{f} = \left \{c\in C\left |[[ K]]^{f}_{c}=1\right .\right \}$

The centered proposition expressed by DRS *K* simpliciter is a special case: [[*K*]]=[[*K*]]^∅^.

This concludes our crash course in DRT syntax and semantics. Now let’s return to the syntax and semantics of ADSs.

### Towards a Syntax and Semantics of ADT

An ADS contains descriptions of various mental files and attitudes in the form of labeled DRSs (boxes). We’ll need to decide how to line up these components of the ADS with the attitude sets provided by the model. The idea is to match the bel-labeled DRS with the set of doxastic alternatives, the des-labeled DRS with the buletic alternatives, etc. We run into a number of complications here, so to keep things manageable I’ll suggest some effective simplifications.

The first complication is that Kamp’s ADSs may contain various, distinct bel- or des-labeled DRSs, but the Kripke/Hintikka model provides only one set of doxastic alternatives for an agent (at a given time and world). One of the reasons for allowing multiple belief boxes is to address the problem of logical omniscience using the fragmentation strategy of Lewis (1982): a mental state can contain distinct belief compartments, which, taken together would be inconsistent. I will simply ignore this orthogonal issue here and assume that an ADS contains at most one DRS per attitude label (cf. Maier 2015c for a semantics that does take into account fragmentation).

We can now use convenient notations like *K*_IMG_ to refer to the img-labeled DRS component of an ADS $\mathcal {K}$. For concreteness, let’s further restrict attention to the attitudes of belief, desire, and imagination. In other words, we’ll consider only ADSs of the following form:

(14) $\mathcal {K} = \left \{\begin {array}{c}\left \langle \textsc {anch},K_{\textsc {anch}}^{1}\right \rangle , \ldots , \left \langle \textsc {anch},K_{\textsc {anch}}^{n }\right \rangle \\ \langle \textsc {bel},K_{\textsc {bel}}\rangle , \langle \textsc {des},K_{\textsc {des}}\rangle , \langle \textsc {img},K_{\textsc {img}} \rangle \end {array}\right \}$

One final restriction is that I will focus only on dependencies involving anchors, ignoring potential dependencies between attitudes.^6^ With these restrictions in place we can already define the wide contents of the various attitudes in an externally anchored ADS $\langle \mathcal {K},f\rangle $ (where $\mathcal {K}$ is an ADS of the form in (14) and *f* an external anchor that maps all internally anchored discourse referents in $\mathcal {K}$ to elements in the domain *D* of the model). The belief content expressed by $\langle \mathcal {K},f\rangle $ is simply the proposition expressed by the bel-labeled DRS in $\mathcal {K}$, relative to external anchor *f*, as defined in standard DRT (cf. (13)): [[*K*_bel_]]^*f*^. The same holds for the other attitudes. The wide content of my hope in the letter example is then the set of centered worlds in which the actual letter, the causal source of my internal anchor, is a notification of acceptance. Pierre’s wide belief content is the set of worlds *w* such that the actual city, London, is pretty in *w* and not pretty in *w*, i.e., the empty set.

Note that internal anchors and the attitudes provided by the model play no role in the definition of wide content; (14) just defines the singular propositions actually expressed by mental states. Psychologically speaking, whether or not $\mathcal {K}$ accurately describes someone’s mental state (given by the model) has nothing to do with such singular propositions. As announced in Section 4.2, to capture the psychological interpretation (that can explain for instance the agent’s behavior) we need a different notion of attitude content: narrow content. In determining, say, narrow desire content, the free discourse referents in the desire box should get their reference fixed not by the external anchor, but by the descriptive content in the internal anchors.

Internal anchors introduce some further complications. First of all, what attitude should we associate with anch? That is, what attitude – if any – does an agent have toward the content of her mental files. From the three attitudes considered so far, belief is the most promising candidate. If an internal anchor contains the descriptive condition ‘see(i,x)’ or ‘named(x, *Londres*)’ this entails that I believe that I’m seeing x, or that x is called ‘Londres’. The interpretation of internal anchors as beliefs is consistent with the views of both Recanati, as brought out in the discussion with Ninan reconstructed in Section 1, and Kamp et al. (2003).^7^ Now, the first complication comes up again: there are multiple anchors and only one set of belief alternatives. To get around this I will just assume that all internal anchors and the belief box merged together describe things that the agent believes to be the case.^8^

We are now in a position to formulate the central definition of ADT semantics a bit more precisely. The idea is to use the intensional DRT semantics to compute the propositions expressed by the merged belief-plus-anchors DRS (notation: $K^{1}_{\textsc {anch}} \oplus {\ldots } \oplus K^{n}_{\textsc {anch}} \oplus K_{\textsc {bel}}$), the desire DRS (*K*_DES_), and the imagination DRS (*K*_img_), and then compare those propositions with the sets of doxastic, buletic and imaginative alternatives in the usual Hintikka/Kripke fashion:

(15) $\begin {array}{lll} \text {An ADS}~\mathcal {K} \text {captures an agent \textit {a}'s mental state in \textit {w} iff}~\\ \text {a.}{\kern 4pt} Dox(a,w)\subseteq [[ K^{1}_{\textsc {anch}} \oplus {\ldots } \oplus K^{n}_{\textsc {anch}} \oplus K_{\textsc {bel}}]]\\ \text {b.}{\kern 4pt} Bul(a,w)\subseteq [[ K_{\textsc {des}}]]{\kern 45pt} [\text {to be revised}]\\ \text {c.}{\kern 4pt} Img(a,w)\subseteq [[ K_{\textsc {img}}]]{\kern 43pt}[\text {to be revised}] \end {array}$

For some very simple examples where all non-doxastic attitude components in an ADS are self-contained, proper DRSs this will give adequate results. However, the whole point of the ADS structure is to allow referential dependencies between attitudes, which involves attitude DRSs with free variables. In the letter example, for instance, we saw the singular hope that it – the letter – is a notification of acceptance. Formally, the desire DRS in that example contains the free variable x and hence does not express a proposition: [[*K*_DES_]] in (15b) is undefined.

In sum, the classical proposition-based approach fails because attitudes that are referentially dependent on other attitudes do not express propositions. There are several solutions to this problem. Kamp’s own proposal, for instance, uses a dynamic rather than static DRT semantics, because that allows him to assign appropriate semantic values to DRSs with free variables (cf. Kamp et al. 2003). Below I will present a simpler, more conservative, but less general solution for our (already stripped down) ADS syntax. Nonetheless, the proposal below is still expressive enough to deal with all the philosophically interesting cases discussed above, as well as Ninan’s example discussed in Section 1. For a more comprehensive semantic treatment of ADSs with arbitrary numbers of distinct, possibly conflicting belief fragments, as well as arbitrary chains of dependencies between any number and type of attitudes in an ADS, I refer the reader to Maier (2015c).^9^

The problem with (15) was that we could have free variable in *K*_DES_ and *K*_img_. Note first that we don’t have this problem with belief. The free variables in a belief box are taken care of by merging the belief with the anchors in (15a). This simple fix won’t work for other attitudes because merging the anchors in (15b) would entail that the agent *wants* the contents of her mental files to be satisfied.

What we need is an external anchor to fix the reference of these free imagination/desire variables “in the background”. But where do we get such an external anchor from? Answer: we construct it from interpreting the material in the internal anchors. In other words, we want to evaluate the desire and imagination components of the ADS relative to embeddings and doxastic alternatives that verify the conditions in the internal anchors.

Anticipating Ninan’s example, a proper implementation of this idea requires that we replace *Bul* and *Img* with two-dimensional or “parasitic” notions of desire and imagination, *Bul*^∗^ and *Img*^∗^. Following a suggestion from Ninan (2008) two-dimensional attitudes specify the agent’s buletic and imaginative alternatives only relative to a centered world the agent believes to inhabit. More precisely, *Img*^∗^(*a*, *w*, *c*), say, is the set of worlds compatible with what the agent *a* in *w* would imagine if her belief set were the singleton {*c*}, i.e. if she were convinced she was *a*_*c*_ inhabiting *w*_*c*_ without any uncertainty (cf. Ninan 2008:43–44 for more detailed motivation for such a primitive two-dimensional notion of imagination).

The definitive narrow psychological semantics for ADT can now be stated as follows:

(16) $\begin {array}{lll} \text {An ADS} \mathcal {K} \text {captures the mental state of \textit {a} in~} w~ \text {iff}\\ \text {a.}~{\kern 4pt} Dox(a,w)\subseteq [[ K^{1}_{\textsc {anch}} \oplus {\ldots } \oplus K^{n}_{\textsc {anch}} \oplus K_{\textsc {bel}}]]\\ \text {b.}~ \text {for all~} c\in Dox(a,w)\,\text {and all} f \text {with}\,Dom(f)= U(K^{1}_{\textsc {anch}} \oplus {\ldots } \oplus K^{n}_{\textsc {anch}})\\ {\kern 8pt} \text {and}\,f\models _{c} K^{1}_{\textsc {anch}} \oplus {\ldots } \oplus K^{n}_{\textsc {anch}} \text {~and}\, f(\textnormal {i})=a_{c}:\\ {\kern 8pt} \text {(i)}~Bul^{*}(a,w,c)\subseteq [[{K_{\textsc {des}}}]]^{f}\\ {\kern 8pt}\text {(ii)}~Img^{*}(a,w,c)\subseteq [[{K_{\textsc {img}}}]]^{f} \end {array}$

Applied to the letter example (i.e., (5), but minus the intention), $\mathcal {K}$ captures *a*’s mental state given by *Dox* and *Bul*^∗^ iff the following two conditions hold: (a) $Dox(a,w)\subseteq [[{K^{1}_{\textsc {anch}} \oplus {\ldots } \oplus K^{n}_{\textsc {anch}} \oplus K_{\textsc {bel}}}]]$, i.e., for every doxastic alternative *c*∈*Dox*(*a*, *w*) there is an embedding *f* such that, in *c*: *f*(x) is an envelope, *f*(y) a mailbox, *f*(x) is in *f*(y), the belief center *a*_*c*_ sees *f*(x) and *f*(y) in front of her, and *f*(x) is junk mail. In other words: the agent believes she’s seeing a piece of junk mail in an envelope in her mailbox. The second condition, (b), says that for every doxastic alternative *c* and every *f* that verifies the merged internal anchor DRS in *c* (i.e., every *f* with *f*(x) is an envelope that *a*_*c*_ sees in a mailbox *f*(y) in front of her in *c*): *Bul*^∗^(*a*, *w*, *c*) ⊆ [[*K*_DES_]]^*f*^, i.e., *f*(x) is a notification of acceptance in all buletic alternatives of the agent, relative to the background assumption of the agent inhabiting that doxastic alternative *c*. In other words, (b) says that, relative to each doxastic alternative *c*, with relevant letter *f*(x), the agent wants *f*(x) to be a notification of acceptance. Taken together, (a) and (b) adequately capture the belief and hope described in the scenario: the agent believes the letter she’s looking at is a piece of junk mail, but, hopes that it, the letter she believes she’s seeing, is a notification of acceptance.

## Ninan’s Objection Revisited

Now let’s revisit Ninan’s challenge to Recanati and see how our ADT deals with it. As you’ll recall, Lucy is acquainted with Samuel Clemens, the writer, in two different ways. On Recanati’s picture, she has two co-referential mental files, one on Clemens, containing the information that he bears that name and is not famous, and one on Twain, containing the information that he bears that other name and is a famous author. In ADT we represent Lucy’s mental state at this point as containing two internal anchors, externally anchored to the same individual, introducing two mental discourse referents.^10^ Assuming that the property of being famous or not famous is not strictly part of the acquaintance relation – the example doesn’t specify the exact status of this information – we might represent that information in a belief box rather than add it to the anchor.^11^

(17)

The ADS in (17) is a straightforward representation of this Fregean double vision scenario. On the one hand, the wide content expressed by the belief box is the inconsistent proposition that Clemens is both famous and not famous. On the other hand, the ADS in (17) captures Lucy’s narrow, psychological mental state (in *w*) iff in all her doxastic alternatives there are two entities of which one is named *Twain* and is famous, and the other is named *Clemens* and is not famous – which is not at all inconsistent.

Now, the challenge involves Lucy imagining contrary to what she believes that it were Clemens who is famous instead of Twain. Since we represent imagination separate from belief this should pose no problem:

(18)

To be sure that this formula indeed makes sense we have to check its narrow semantic interpretation. The ADS in (18) captures Lucy’s mental state iff (a) all her doxastic alternatives have a verifying embedding mapping x to a famous Twain and y to a non-famous Clemens, as above, and (b) for all doxastic alternatives *c* and all anchor-verifying-embeddings *f* (i.e. with *f*(x) is named *Twain* in *c* and *f*(y) is named *Clemens* in *c*): *Img*^∗^(Lucy, *w*, *c*) ⊆ [[*K*_img_]]^*f*^. The (b) requirement boils down to the condition that in all of Lucy’s imagination alternatives, relative to each of her doxastic alternatives, *f*(x), the doxastic alternative’s Twain, is not famous, but *f*(y), the doxastic alternative’s Clemens, is. Again, there’s no inconsistency, and the interpretation seems to capture precisely what’s going on in Lucy’s head, using only the tried and tested formal machinery of possible worlds semantics.

In closing let me address one potential objection. An observant reader may well object that the reason we avoided inconsistency is that already in (17) we chose to represent the properties of being famous or not famous in the belief box rather than as part of the internal anchor, contrary to what Recanati would have done in his mental file framework. To drive this point home she could then point to Ninan’s earlier puzzle about counterfactual *de re*, where we clearly have an imagination that is inconsistent with the information represented in the internal anchor (Ninan 2008, 2012). For instance, Ralph imagines *de re* of the individual he is now seeing for the first time, what it would be like if he would never have seen him (or what it would be like if this guy had never even existed).

As it turns out the syntax and semantics provided above deals with such trickier cases as well – in fact, I incorporated one of Ninan’s own solutions, the primitive two-dimensional notion of belief-relative-imagination, for just this purpose. Consider a minimal representation of Ninan’s (2008) puzzle:

(19)

Fully in line with the description of the case, our semantics says that (19) captures Ralph’s (narrow) mental state if he believes there to be someone he is seeing, and relative to all his belief alternatives *c* (containing at least one individual *f*(x) he is seeing there (and then)), he imagines that he is not seeing *f*(x).

## Conclusion

I have presented a DRT-based formal theory for the representation of complex mental states. It analyzes singular attitudes via descriptive internal anchors that are externally anchored to objects in the world. Much like Recanati’s mental files, ADT’s anchors are, as Ninan puts it, “mental representations whose primary function is to carry information about objects”.

The crucial difference between Kampian ADSs and Recanati’s mental files is that an ADS represents the different attitudes (belief, desire, imagination) separately, as distinct DRS boxes that all have access to the discourse referents introduced by the anchors. In this paper I have shown that the resulting framework is expressive enough to describe mental states involving doxastic and non-doxastic attitudes, *de se* attitudes, double vision, hallucination, vicarious anchors, and, finally, Ninan’s counterfactual attitudes.

## Footnotes

1 The original semantics of DRT is static, so its dynamic aspect lies wholly in its construction algorithm, i.e., the way it turns sentences into DRS updates. Later presentations of the framework also add a dynamic semantics in terms of Heimian context change potentials.

2 I do not distinguish between different buletic attitudes such as hoping, wanting, and desiring. I label them all des, and interpret them in terms of a set of so-called *buletic alternatives*.

3 Exactly what kind of information counts as representing a relation of acquaintance between subject and *res* and thus belongs in an internal anchor is a delicate matter that I will return to in Section 4.3 below.

4 Equivalently, we can think of an attitude DRS *K* containing i as expressing the property expressed by *λ*i.*K*.

5 In recent work, Kamp (2015:286-287) proposes an alternative picture, where an “entity representation” contains both a set of internal anchor DRSs containing acquaintance-based content (like ‘see(i,x)’), and a DRS containing descriptive contents (like ‘philosopher(y)’ or ‘name(x, *John*)’). It is not clear to me at this point what, if anything, is the difference between the belief box in the current proposal and the descriptive part of such a complex entity representation.

6 We could easily add belief dependence to the system below, but desires or beliefs dependent on an imagination are more difficult (and also controversial). This limitation of the current system is one of the primary reasons for introducing much heavier formal machinery in my 2015cproposal.

7 A plausible, weaker alternative I’ve discussed elsewhere in connection with fictional anchors is the view that anchors contain material that the agent *accepts* (Maier 2015a).

8 DRS merge is defined as follows: *K*_1_⊕*K*_2_ = 〈*U*(*K*_1_)∪*U*(*K*_2_), *C**o**n*(*K*_1_)∪*C**o**n*(*K*_2_)〉.

9 The semantics below is based on (Maier 2015b), which is somewhat simpler than the semantics proposed in (Maier 2016). The latter is more fine-grained in that it distinguishes more clearly between internal anchors and beliefs, semantically: internal anchors, but not beliefs, provide enough detail to determine a *unique* embedding verifying all internal anchors relative to any given doxastic alternative. In the current proposal I lump together beliefs and internal anchors and drop that requirement.

10 Probably, these anchors are in fact vicarious, so a more accurate representation would contain information about how she learned about Clemens and Twain. Since the example doesn’t specify, I’m just giving the name condition in the anchors in (17).

11 Note that, with the simplified semantics above, the syntactic choice between putting something in a belief box or an internal anchor ultimately does not really affect the psychological interpretation. This would be different if we would interpret anchors via acceptance or take fragmentation into account.
